# Relationship between plumage color and eggshell patterns with egg production and egg quality traits of Japanese quails

**DOI:** 10.14202/vetworld.2021.897-902

**Published:** 2021-04-14

**Authors:** Ly Thi Thu Lan, Nguyen Thi Hong Nhan, Lam Thai Hung, Tran Hoang Diep, Nguyen Hong Xuan, Huynh Tan Loc, Nguyen Trong Ngu

**Affiliations:** 1 Department of Animal Science and Veterinary Medicine , School of Agriculture and Aquaculture, Tra Vinh University, Tra Vinh City, Vietnam; 2 Department of Animal Science, College of Agriculture, Can Tho University, Can Tho City, Vietnam; 3 Department of Science and Technology, Tra Vinh Province, Tra Vinh City, Vietnam; 4 Department of Animal Science and Veterinary Medicine , Faculty of Agriculture and Food Technology, Tien Giang University, My Tho City, Vietnam; 5 Department of Food Technology, College of Food Technology and Biotechnology, Can Tho University of Technology, Can Tho City, Vietnam; 6 Department of Veterinary Medicine, College of Agriculture, Can Tho University, Can Tho City, Vietnam

**Keywords:** egg yield, feather color, linkage, morphological characteristics

## Abstract

**Aim:**

This study was conducted to identify the diversity of feather color and to determine the relationship between plumage color and egg yield as well as eggshell patterns and internal egg quality traits of Japanese quails.

**Materials and Methods:**

For investigating phenotypic diversity, a total of 600 quails from five breeding farms were evaluated to record head feather, shank, and plumage color. An on-station experiment was also conducted on 360 laying quails to examine the relationship between plumage color and egg production and egg weight during 24 weeks of laying. Eggs collected during this period were also used for identifying eggshell patterns and examining their relationship with internal egg quality characteristics.

**Results:**

Plumage color was primarily wild-type, with the highest proportion being 56.3% (p<0.001). Brown color was also found at a relatively high proportion in the population (16.7%), followed by black color (11.3%). The egg production and laying rate of quails with wild-type and brown plumage colors also significantly (p=0.001) differed from those of quails with other plumage types. Egg weight was also higher in these quail groups, especially than that of quails with yellow plumage color. Four patterns of eggshell were identified, among which spotted and dark eggshells were predominant (45.2% and 43.1%, respectively); however, patterns did not affect internal egg quality characteristics.

**Conclusion:**

Plumage color was primarily wild-type in both male and female quails. Egg yield over a 24-week laying period was superior in quails with wild-type and brown plumage colors, whereas a relationship between eggshell patterns and egg quality traits could not be established.

## Introduction

One of the important characteristics that help in distinguishing poultry species from other vertebrate species is the presence of feathers that provide them the ability to fly, disguise, and regulate their temperature. The change in the color of feathers in poultry has attracted the attention of ecologists and evolutionary biologists. Feather colors have been used predominantly in several areas, including sex selection, analysis of geographic differences and speciation, and the evolution of polymorphism [1]. Most of the color changes in the plumage within and between species have a strong genetic component [2]. Bedhom *et al*. [3] mentioned that the interaction of specific gene mutations or combinations of mutations is a major cause of change in the plumage color of quails. Sezer and Tarhan [4] have also attempted to link the effects of feather color with growth characteristics in Japanese quails, and they concluded that morphological characteristics, specifically feather color, contributed significantly to the identification process as well as the selection of quail varieties.

In addition to plumage color, eggshell and its spot colors are extremely diverse. Sezer and Tekelioglu [5] demonstrated that the color of quail eggshell varies from white to blue or green, whereas Taha [6] classified quail eggs as black-spotted eggs with different sizes on brown- or gray-colored eggshell, spotless white eggs, and eggs with small black or blue spots on gray-brown-colored eggshell. In contrast, Hassan *et al*. [7] classified quail eggs as bright eggs (without spots or very small), dotted eggs (with small spots), spotted eggs (with large spots), and dark eggs (with a few very large spots). The variety of eggshell color in quails has attracted several scientists. Alasahan *et al*. [8] also investigated the relationship between color and spots on eggshell and the internal and external qualities of eggs. Their results showed that eggshell color and color of spots had a significant effect on egg quality characteristics such as eggshell ratio, eggshell index, albumen index, yolk index, and Haugh unit. Other studies have focused on the impact of eggshell color changes on eggshell structure, egg weight reduction, and hatching parameters [6,7], as well as internal and external quality characteristics of eggs.

Japanese quails (*Coturnix coturnix japonica*) have emerged as a research target due to their rapid growth, early sexual maturity, high egg production rate, short reproduction time, minimal requirements of space and food, easy maintenance, high resistance against several diseases in poultry, and potential for meat and eggs [9]. Despite the advantageous dominance over other poultry species, quail-breeding program has not been completely exploited, especially in the Mekong Delta of Vietnam where an approximate number of 3.19 million quails are being raised every year primarily for laying purposes [10]. One of the major issues in our knowledge regarding the selection of breeding quails is a lack of information on the relationship of phenotype with egg yield and other related traits to streamline the production aimed at an optimal profit.

Therefore, the present study was conducted to determine the relationship between plumage color and egg production as well as eggshell patterns and internal egg quality traits. The results of this study may serve as a stepping stone for the selection and hybridization of quails based on phenotypic characteristics and may also provide useful information for selecting eggs using eggshell colors.

## Materials and Methods

### Ethical approval

This study was carried out after obtaining approval from Can Tho University and Tra Vinh Sub-Department of Animal Health.

### Study period and location

The on-station trial was conducted at the experimental farm of Tra Vinh University, Vietnam (9°55′ N, 106° 20′ E) from February to August 2019.

### Identification of phenotypic characteristics of Japanese quails

The phenotypic diversity of Japanese quails raised in the Mekong Delta was investigated. Most of the farm-reared quails are introduced by breeders in the Mekong Delta in vast numbers for restocking purposes. Using the commercial lines of Japanese quails, these quail lines are thereafter generated and maintained by local breeders for egg production. In this study, five quail-breeding farms were included in the survey, among which for each farm, 30 male and 90 female quails were randomly selected to record physical features, including plumage color, head feather color, and shank color, according to the classification method described by Tsudzuki and Wakasugi [11]. A total of 600 quails (150 males and 450 females) were used in this investigation.

### Experimental quails and management

On the basis of the diversity of quail feathers in the survey, quails were obtained from one quail-breeding farm consisting of quails with four popular plumage types, that is, black, brown, wild-type, and yellow, with 90 female quails per group. The laying performance of 360 quails (167±7.8 g) was recorded during 24 weeks of laying. Quails were individually maintained in each cage and provided a commercial diet consisting of 21% crude protein and 2850 kcal of ME/kg from a feed company. Feed and water were available *ad libitum* all time, and a lighting program of 16 h of light/day was applied. Quails were vaccinated against Newcastle disease and infectious bursal disease (Gumboro). For the analysis of external and internal traits, eggs were collected on a given day in the 4^th^, 8^th^, 12^th^, 16^th^, 20^th^, and 24^th^ laying weeks for weighing and quality measurement.

### Egg measurements

In total, 2160 eggs were included for recording egg weight and shell pattern during the experimental period. The categorical classification of the eggshell patterns of Japanese quails was based on Hassan *et al*. [7]. For interior quality characteristics, one-fourth of the eggs (540 eggs) were analyzed. A digital display caliper was used to measure the width, length, yolk diameter, and albumen length and width of the eggs; yolk weight was measured to an accuracy of 0.01 g, and the proportion of this parameter was expressed in percentage corresponding to egg weight. Yolk color was determined using the La Roche scale scoring from 1 to 15 [12]. Albumen weight was calculated by subtracting the yolk and shell weight from the whole egg weight. These data were used to determine the external and internal quality characteristics of eggs using the following formulas [13]: Shape index (%)=(Width [mm]/Length [mm])×100; Shell ratio (%)=(Shell weight [g]/Egg weight [g])×100; Albumen weight (g)=Egg weight−(Shell weight+Yolk weight); Yolk ratio (%)=(Yolk weight [g]/Egg weight [g])×100; Albumen ratio (%)=(Albumen weight [g]/Egg weight [g])×100; Yolk index (%)=(Yolk height [mm]/Yolk diameter [mm])×100; Albumen index (%)=(Albumen height [mm]/[(Albumen length [mm]+Albumen width [mm])/2])×100; Haugh unit=100 log (Albumen height [mm]+7.57−1.7×Egg weight [g]^0.37^).

### Statistical analysis

All data were subjected to statistical analysis using the Minitab 16.2 software (State College, PA, USA) [14]. The Chi-square test was used to evaluate the distribution of morphological characteristics, and a general linear model Y_ij_=μ+W_i_+e_ij_ was used to evaluate the relationship between phenotypes and production and quality traits, where Y_ij_ is the phenotypic value of the traits, μ is the overall mean, W_i_ is the effect of plumage color (black, brown, wild-type, and yellow), and e_ij_ is the random error.

## Results

### Phenotypic characteristics

The feather color of Japanese quails in the Mekong Delta was diverse and distributed differently between female and male quails as well as between body parts as shown in Table-1. Regarding head feather color, yellow accounted for the highest proportion (54.0%) in male quails (p=0.001), whereas in female quails, black plumage color (39.1%, p=0.001) was predominant over other colors, although the proportion of male quails with this color was the lowest (2.0%). Furthermore, gray color was found in a high proportion of male and female quails, with 31.3% and 23.1%, respectively. Moreover, plumage color was primarily wild-type for both male (44.7%) and female (60.2%) quails, with the highest average rate being 56.3% (p=0.001). Brown color was also identified at a relatively high rate in the quail population (16.7%). Coincidentally, black color had the lowest proportion in the population (11.3%). In addition, for shank color, the highest proportion was found for pinkish-brown (62.7%), followed by yellow (24.3%) for both male and female quails. Although orangish-gray color appeared at a high rate (29.3%) in the population of male quails compared with yellow color (3.3%) (p=0.001), it still accounted for the lowest percentage of the total population surveyed.

**Table-1:** Distribution of feather color in breeding Japanese quails raised in the Mekong Delta.

Color	Male (n=150)		Female (n=450)		Total (n=600)	
		
	Number	Ratio (%)	Number	Ratio (%)	Number	Ratio (%)
Head						
Black	3	2.0^d^	176	39.1^a^	179	29.8^ab^
Black stripes	19	12.7^c^	41	9.1^d^	60	10.0^c^
Gray	47	31.3^b^	104	23.1^c^	151	25.2^b^
Yellow	81	54.0^a^	129	28.7^bc^	210	35.0^a^
p-value		0.001		0.001		0.001
Plumage						
Black	26	17.3^b^	42	9.3^d^	68	11.3^c^
Brown	36	24.0^b^	64	14.2^c^	100	16.7^b^
Wild-type	67	44.7^a^	271	60.2^a^	338	56.3^a^
Yellow	21	14.0^b^	73	16.2^bc^	94	15.7^bc^
p-value		0.001		0.001		0.001
Shank						
Orangish-gray	44	29.3^b^	34	7.6^c^	78	13.0^c^
Pinkish-brown	101	67.3^a^	275	61.1^a^	376	62.7^a^
Yellow	5	3.3^c^	141	31.3^b^	146	24.3^b^
p-value		0.001		0.001		0.001

^a,b,c,d^ Means bearing different superscripts within a column differ significantly (p<0.05)

### Relationship between plumage color and egg production

Egg production was found to have a close relationship with plumage color (Table-2). During all the recorded laying periods, a significant difference (p=0.001) was observed between the types of brown and wild-type plumage colors and the other types of black and yellow colors in the laying weeks. In general, there were significant differences in the two types of brown and wild-type plumage colors on the average number of eggs (150.4 and 155.4, respectively) and laying rate (89.5% and 92.5%, respectively). Egg weight was also higher in these quail groups, especially in quails with the yellow plumage color. Furthermore, although the mean shape index significantly differed in the 12^th^ week between all types of quails, the average value observed during the 24 weeks of laying was similar, ranging from 75.6% to 76.7%.

**Table-2:** Relationship between plumage colors and egg production traits.

Parameters	Plumage color	Laying weeks						

		1-4	5-8	9-12	13-16	17-20	21-24	1-24
Egg production	Black	17.2±0.54^b^	21.2±0.38^b^	22.6±0.42^b^	22.4±0.49^b^	21.2±0.47^b^	19.1±0.65^b^	124.8±1.54^b^
	Brown	20.3±0.54^a^	24.0±0.36^a^	26.5±0.39^a^	26.6±0.46^a^	26.5±0.44^a^	26.4±0.61^a^	150.4±1.45^a^
	Wild-type	22.1±0.54^a^	24.8±0.37^a^	27.4±0.40^a^	27.3±0.47^a^	26.9±0.45^a^	26.8±0.62^a^	155.4±1.48^a^
	Yellow	17.7±0.54^b^	21.3±0.41^b^	22.4±0.44^b^	20.7±0.52^b^	21.2±0.49^b^	19.0±0.69^b^	122.1±1.64^b^
	p-value	0.001	0.001	0.001	0.001	0.001	0.001	0.001
Laying rate (%)	Black	59.9±2.03^b^	75.5±1.36^b^	81.2±1.49^b^	79.8±1.77^b^	75.7±1.69^b^	68.3±2.32^b^	74.3±0.92^b^
	Brown	72.7±1.90^a^	85.6±1.27^a^	94.8±1.34^a^	95.1±1.65^a^	94.6±1.58^a^	94.1±2.17^a^	89.5±0.86^a^
	Wild-type	79.2±1.95^a^	88.7±1.30^a^	97.9±1.43^a^	97.5±1.67^a^	95.9±1.61^a^	95.8±2.22^a^	92.5±0.88^a^
	Yellow	62.7±2.14^b^	76.2±1.43^b^	80.5±1.57^b^	74.3±1.86^b^	75.2±1.78^b^	68.7±2.45^b^	72.6±0.97^b^
	p-value	0.001	0.001	0.001	0.001	0.001	0.001	0.001
Egg weight (g)	Black	11.3±0.12^b^	11.7±0.10^ab^	11.9±0.10	11.7±0.11^ab^	11.5±0.15	11.7±0.10	11.6±0.09^ab^
	Brown	11.7±0.12^a^	12.0±0.09^a^	12.0±0.10	12.0±0.10^a^	11.8±0.14	12.0±0.10	11.9±0.08^a^
	Wild-type	11.8±0.12^a^	11.9±0.10^ab^	11.9±0.10	11.9±0.10^a^	11.6±0.14	11.9±0.10	11.8±0.09^a^
	Yellow	11.3±0.13^b^	11.6±0.11^b^	11.7±0.11	11.5±0.11^b^	11.3±0.16	11.6±0.11	11.5±0.10^b^
	p-value	0.002	0.018	0.128	0.011	0.057	0.096	0.004
Shape index (%)	Black	75.1±0.72	74.5±1.17	74.8±0.59^b^	74.9±0.72	74.1±1.03	75.9±0.34	75.6±0.43
	Brown	77.1±0.66	76.2±1.07	77.3±0.58^a^	76.5±0.65	75.1±0.94	76.7±0.30	76.7±0.42
	Wild-type	76.0±0.68	75.3±1.11	77.1±0.58^a^	76.3±0.68	75.1±0.97	76.7±0.32	76.2±0.42
	Yellow	76.0±0.76	73.6±1.23	74.7±0.60^b^	75.6±0.75	74.0±1.08	76.8±0.35	75.6±0.43
	p-value	0.233	0.439	0.001	0.396	0.764	0.220	0.237

^a,b^ Means bearing different superscripts within a column differ significantly (p<0.05)

### Effects of eggshell patterns on egg quality characteristics

In terms of eggshell patterns, spotted and dark eggs accounted for a higher proportion (45.2% and 43.1%, respectively) than bright and little-spotted eggs (Table-3); however, the patterns did now show any relationship with internal egg quality traits (p>0.05).

**Table-3:** Internal quality characteristics of eggs from different eggshell patterns.

Parameters	Eggshell patterns				p-value

	Bright	Dotted	Spotted	Dark	
Number of eggs	108	145	976	931	
Proportion	5.0^d^	6.7^c^	45.2^a^	43.1^ab^	0.001
Egg characteristics					
Shell weight (g)	1.68±0.05	1.62±0.04	1.64±0.02	1.62±0.02	0.649
Shell ratio (%)	14.9±0.45	14.2±0.39	14.2±0.15	14.3±0.15	0.542
Yolk weight (g)	3.51±0.08	3.58±0.07	3.59±0.03	3.58±0.03	0.873
Albumin weight (g)	6.15±0.15	6.3±0.13	6.30±0.05	6.17±0.05	0.216
Yolk ratio (%)	31.0±0.66	31.2±0.57	31.1±0.22	31.5±0.22	0.591
Albumin ratio (%)	54.0±0.96	54.7±0.83	54.6±0.32	54.2±0.33	0.797
Yolk/albumin ratio (%)	58.8±2.02	58.6±1.75	57.7±0.67	58.8±0.70	0.722
Yolk index (%)	40.9±1.12	40.9±0.97	40.6±0.37	40.3±0.38	0.907
Albumin index (%)	9.63±0.43	9.53±0.37	9.10±0.14	9.18±0.15	0.516
Haugh unit	88.7±0.84	88.9±0.72	87.5±0.28	87.7±0.28	0.201

^a,b^ Means bearing different superscripts within a row differ significantly (p<0.05)

## Discussion

Japanese quails in the Mekong Delta were found to be quite diverse in terms of plumage colors. Mishra *et al*. [15] also reported that Japanese quails had breast colors such as white, brown, and white brown, wherein the presence of white color was due to a new mutation of dominant gene activity at a locus in the genome. According to a previous study conducted by Tsudzuki and Wakasugi [11], the plumage of quails consists of colored bands arranged from tail to head with some common colors such as gray, black, white-gray, brown, wild-type, and yellow. Wild-type was found to be the most popular plumage color in the quail population, and different feather colors were also identified between male and female quails. The dominant population of wild-type quails can be explained by the fact that the wild-type group had lower mortality rates and higher values of internal and external quality characteristics of eggs than others, including the white, dark brown, and golden plumage color groups [16]. Another study reported that wild-type Japanese quails had significantly higher slaughter weight than quails of light brown type [15]. Although some studies have shown that white quails had the heaviest body weight with the best carcass traits and meat quality [17-19], this color was not found in the present study. Altogether, it is necessary to consider the favorable characteristics of wild-type quails to improve quail production in the Mekong Delta.

The differences in some phenotypic traits of quails, including egg production, laying rate, egg weight, and shape index, from the four types of plumage colors were also determined in this study. Interestingly, they exhibited a close relationship with egg yield during the 24-week laying period. The values of these traits for quails with brown and wild-type plumage colors were consistently higher than those for quails with black and yellow plumage colors. Supporting this finding, egg production was found to differ significantly according to different quail phenotypes [20,21]. Egg weight was also affected by the diversity of plumage colors, where eggs laid by quails with yellow plumage were lower in weight than those laid by quails with other plumage patterns. In the present study, the brown Japanese quail was superior in terms of egg weight, which is consistent with some previously reported studies [21-23]. These findings support the results obtained by Ashok and Reddy [24] who reported that there were significant differences in egg weight between various types of quails. Overall, these results may be beneficial to select appropriate breeding programs for egg-laying quails in the long term.

Four eggshell color groups were recorded in the present study, among which spotted and dark eggs predominated in the population; these findings were consistent with those of Sezer and Tekelioglu [5]. Basically, egg color forms when it travels through the hen’s oviduct. When the eggshell begins to form, epithelial cells at the surface of the eggshell gland (uterus) begin to synthesize color pigments [25]. The previous studies [26,27] have also confirmed that eggshell color is a genetic transformation of pigments on the egg surface in the uterus. In addition, Alasahan *et al*. [28] classified the color of eggshells into five different groups, including black-spotted gray-white, green-spotted gray-white, scattered brown-spotted gray-brown, brown-spotted light-green, and small brown-spotted gray-brown. However, they also indicated that the effect of eggshell color on egg weight, eggshell mass, albumen weight, and yolk weight was not statistically significant. It was also pointed out that the shell color of Japanese quail eggs, blue or spotted, did not appear to affect their quality [29]; however, it can affect the hatching results and body weight of the obtained chicks of Japanese quails. Nevertheless, Taha [6] reported higher egg weight, egg length, and egg width in the black-spotted quail group than in the blue-spotted quail group. It is worth noting that the determination of the best type of eggshell colors in terms of egg quality is controversial among the published data. In the present study, although spotted and dark eggshells were predominant patterns, the relationship between eggshell patterns and internal egg quality traits could not be established.

## Conclusion

The plumage color of Japanese quails raised in the Mekong Delta of Vietnam was primarily wild-type in both male and female populations. The differences in these color patterns affected egg yield over a 24-week laying period, with superior values in quails with wild-type and brown plumage colors, but no relationship was identified between eggshell patterns and egg quality traits.

## Authors’ Contributions

LTTL, NTHN, and NTN designed the experimental procedures. LTTL, NTHN, LTH, THD, and NHX performed the experiments. LTTL, NTHN, HTL, and NTN interpreted the data and prepared the manuscript. All authors read and approved the final manuscript.
